# The use of Sonidegib in the adjuvant and advanced phases of Sonic Hedge Hog Mutant Medulloblastomas

**DOI:** 10.1093/omcr/omac019

**Published:** 2022-03-16

**Authors:** Alexander K Yuile, Marina Kastelan, Adrian PS Lee, Michael Back, James Drummond, Helen R Wheeler

**Affiliations:** Medical Oncology, Royal North Shore Hospital, Sydney 2065, Australia; The Brain Cancer Group, North Shore Private Hospital, Sydney 2065, Australia; Medical Oncology, Royal North Shore Hospital, Sydney 2065, Australia; Radiation Oncology, Royal North Shore Hospital, Sydney 2065, Australia; Radiology, Royal North Shore Hospital, Sydney 2065, Australia; Medical Oncology, Royal North Shore Hospital, Sydney 2065, Australia

## Abstract

Medulloblastomas are rare embryonal primary brain tumours originating in the cerebellum. Most medulloblastomas arising in adults are associated with mutations in the Sonic Hedge Hog (SHH) pathway. Patient 1 was prescribed Sonidegib for recurrent metastatic SHH mutated medulloblastoma multiple lines of treatment. His leptomeningeal disease responded after 3 months of therapy. The drug was continued for a further 3 months until progressive central nervous system (CNS) and leptomeningeal disease arose. Progression free survival (PFS) from initiation of Sonidegib of 3 months was observed (overall survival 8.8 years). Patient 2 presented with un-resectable SHH mutated meduloblastoma with high risk of relapse who received 14 months of adjuvant Sonidegib. Following biopsy she was treated with chemotherapy and cranio-spinal radiotherapy, followed by 14 months of adjuvant Sonigedib. She remains free of disease over 51 months later. Both clinical scenarios are poorly described in the literature or evaluated in clinical trials with Sonidegib.

## INTRODUCTION

Medulloblastomas are highly cellular embryonal malignant brain tumours presumed to originate from neuronal precursor cells exclusively in the cerebellum. Previous histological classifications have been superseded by molecular classification with four distinct molecular subtypes that carry variable prognosis. These subtypes include anomalies in Wingless-related integration site (Wnt), Sonic Hedge Hog (SHH) pathway, group 3 and group 4 [1]. Although predominantly diagnosed in paediatric patients, medulloblastomas can occur in adults (<1% of adult brain tumours) [2]. The majority of adult medulloblastomas (62%) are driven by mutations in the SHH pathway [2].

SHH mutations are thought to be oncogenic via induction of tumour cell proliferation pathways. SHH binds to the Protein patched homolog 1 (PTCH1) receptor which prevents smoothened (SMO) binding in its place. The unbound SMO then activates downstream pathways stimulating cell growth. The most common mutation leading to this aberrant pathway occurs in PTCH1 but can also be associated with activating SMO mutations [3, 4].

Medulloblastoma management involves a multimodal approach combining maximal safe surgicalx resection, adjuvant cranio-spinal radiotherapy with a boost to the tumour bed and multiagent chemotherapy regimens such as ifosfamide, cisplatin and etoposide (ICE) [5–7]. Meta-analysis showed this combination approach significantly improves overall survival (108 months vs 57 months), but at the cost of significant treatment related toxicities [8].

Recent development of SHH inhibitors (Sonidegib and Vismodegib), which inhibit activated SHH pathways by directly antagonizing SMO [9], offer an emerging novel therapeutic option. These agents target the activated SHH oncogenic pathway in adult medulloblastomas and have the additional potential benefit of significantly less toxicities compared to established treatment protocols. A recent meta-analysis of early phase clinical trials in patients with SHH driven medulloblastoma showed a pooled objective response rate of 37% from treatment with either of the SHH inhibitors [10]. Unlike the case series we present, the patients included in this meta-analysis were neither heavily pre-treated (Case 1) nor treated with SHH inhibitors in the adjuvant setting (Case 2). Furthermore the majority of the data of sonidegib in medulloblastoma (including the above meta-analysis) is from the paediatric setting. This is contrast to this case reports which describes Sonidegib in the adult setting.

## CASE 1

**Figure 1:**
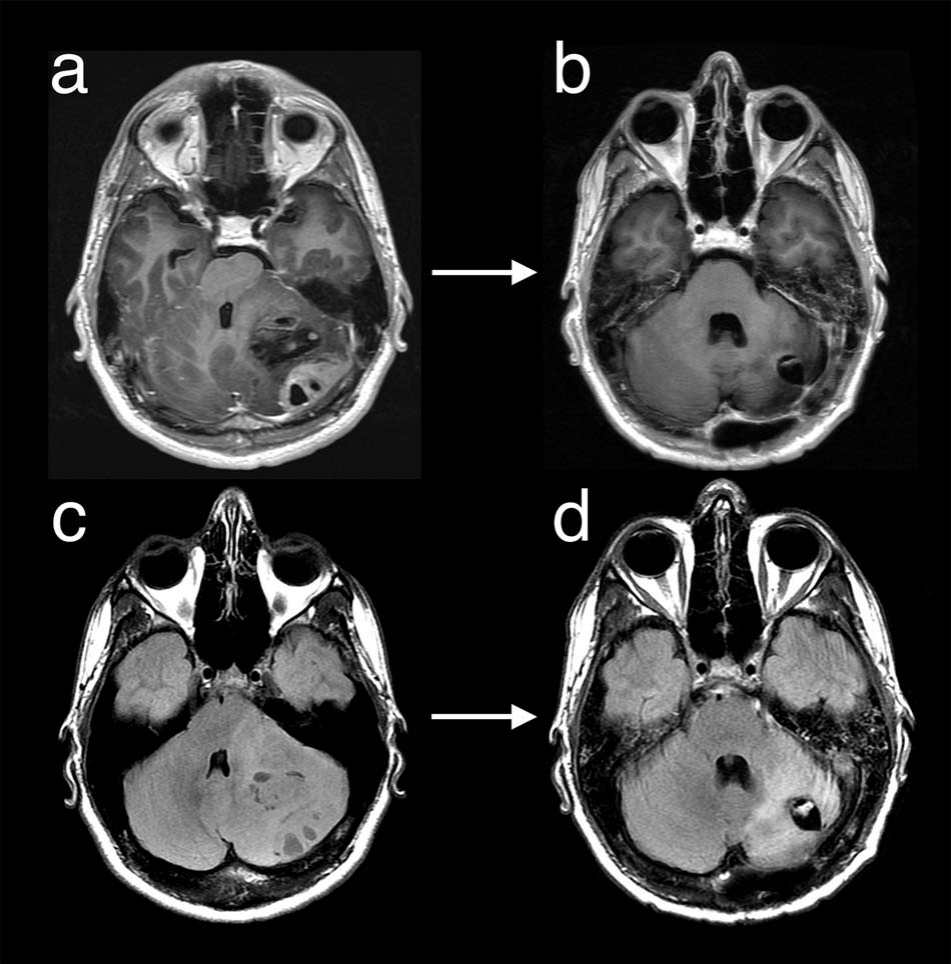
MRI with Axial T1 post-contrast (a/b) and Axial T2 FLAIR (c/d) demonstrate a large heterogeneously enhancing mass centred in the left cerebellar hemisphere with significant mass effect at diagnosis (a/c). After initial therapy with surgical debulking, ICE chemotherapy and cranio-spinal radiotherapy there was no residual enhancing disease (b) with significant reduction in non-enhancing T2 FLAIR signal (d).

A 21-year-old male presented with headaches and nausea in September 2010. Magnetic resonance imaging (MRI) revealed a mixed complex cystic/solid T2 FLAIR hyperintense, heterogeneously enhancing lesion occupying most of his left cerebellar hemisphere (Fig. 1) resulting in significant mass effect and upstream hydrocephalus (Note all MRI studies in this report were performed on a 3.0 T 60 cm bore MR scanner (Discovery MR750, GE Healthcare, US) or 3.0 T 70 cm bore MR scanner and a standard dose (0.1 mmol/kg, 0.2 ml/kg) of gadobenate dimeglumine was used for contrast imaging).

Following surgical debulking, histopathological classification confirmed the ‘classical’ type of medulloblastoma with local leptomeningeal involvement. Spinal MRI demonstrated no radiological evidence of leptomeningeal involvement at initial diagnosis, and three cerebrospinal fluid (CSF) examinations found no abnormal cytology. The patient received three cycles of ICE chemotherapy followed by cranio-spinal irradiation, 23.4 Gy in 13 fractions to spine, 36 Gy to whole brain and a total 59.4 Gy to the surgical bed. MRI brain at completion of chemoradiotherapy showed no residual enhancing disease on T1 post-contrast imaging and 88% volume reduction in the remaining non-enhancing hyperintense disease on T2 FLAIR imaging when compared to preoperative imaging (Fig. 1). After initial treatment, the patient had good functional status and was able to return to work as a landscape gardener.

He remained in remission for 6 years, initially monitored with 3 monthly, then 6 monthly MRI scans. However, in September 2016 a 6 mm enhancing nodule appeared within the surgical bed which gradually increased in size over the following 4 months.

It was surgically resected in February 2017 and pathology revealed recurrent medulloblastoma with desmoplastic/nodular histological features. Molecular sequencing of this recurrent nodule revealed an activating mutation in the SMO pathway (PTCH1 p.516del NM_000264.3 c.1546_1548del), with biallelic loss of TP53. Further clinical staging, including CSF examination excluded disseminated CSF spread.

He received three further cycles of ICE chemotherapy with stem cell harvesting and then proceeded to tandem autologous bone marrow transplants in mid May 2017, four months after re-resection. The first transplant was conditioned with high dose carboplatin, thiotepa and etoposide and second with cyclophosphamide and melphalan. He recovered well and was again able to return to work.

**Figure 2:**
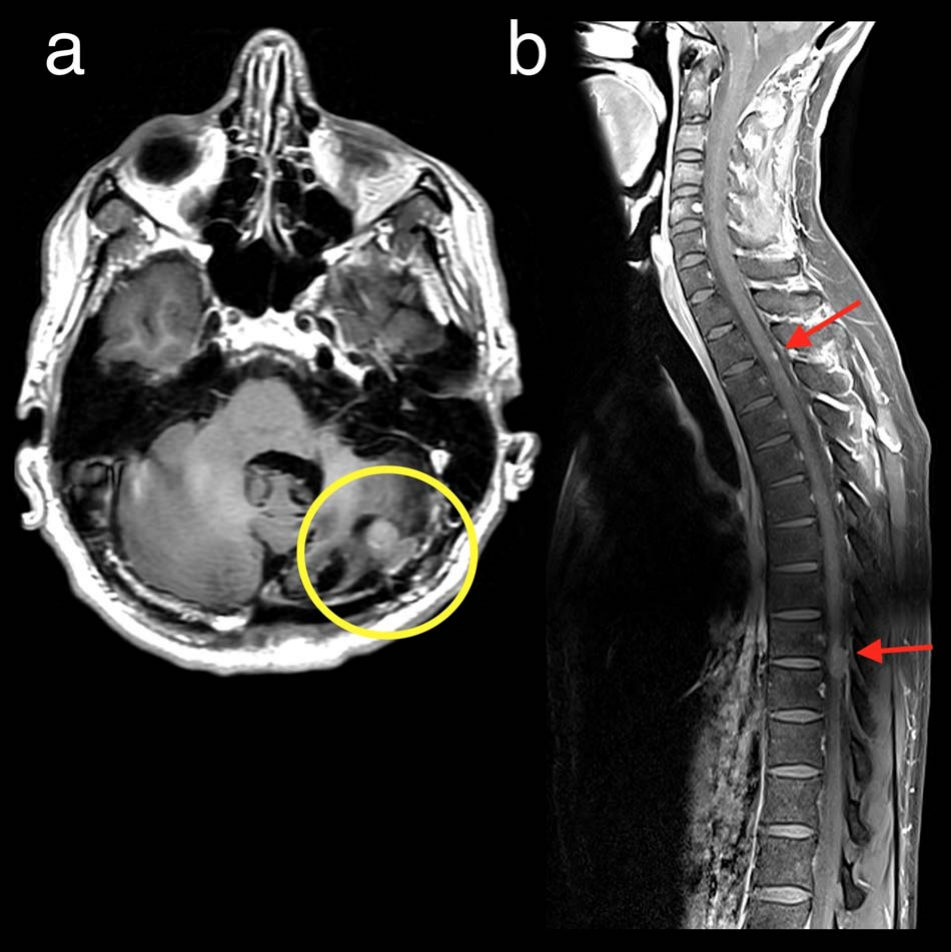
Axial T1 post-contrast (a) and Sagittal T1 post-contrast FS cervico-thoracic spine (b) on follow-up imaging demonstrates recurrent nodular disease in the surgical bed (yellow circle) and new leptomeningeal along the surface of the spinal cord (red arrows).

In May 2018, 12 months post-transplant, a restaging MRI of brain and spine revealed local recurrence in the posterior fossa and new T9/10 nodular enhancement (Fig. 2). He received further radiotherapy to the thoracic spine and posterior fossa totaling 35 Gy in 10 fractions.

**Figure 3:**
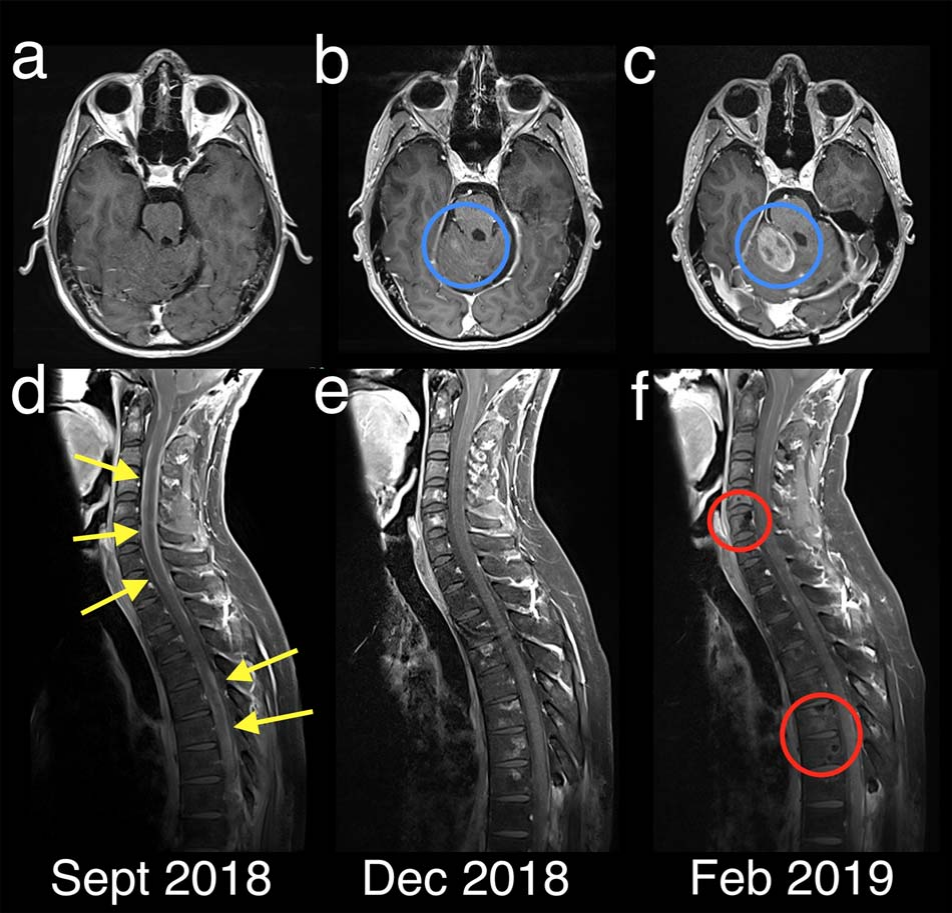
MRI brain and whole spine with Axial T1 post-contrast (a–c) and sagittal T1 post-contrast FS (d–f) following commencement of Sonidegib. In September 2018 (a/c) there was evidence of severe leptomeningeal spread along the spinal cord (yellow arrows) which initially improved on Sonidegib therapy. However, over time there was marked progression of intracranial disease (blue circles) and increased bony metastatic disease (red circles) which preceded further intracranial leptomeningeal spread prior to transition to palliative care.

In September 2018, 4 months post re-irradiation, an MRI whole spine confirmed leptomeningeal spread of the disease and progression at the previously irradiated thoracic spine, as well as bone metastases (Fig. 3); the patient was commenced on Sonidegib with an initial dose of 200 mg daily. It was up-titrated to 600 mg over 2 months, however the patient developed Common Terminology Criteria for Adverse Events (CTCAE) grade 2 diarrhoea and Sonidegib was withheld. Following a 2 week wash out, Sonidegib was reinitiated again at the lower dose of 200 mg daily. By December 2018, the leptomeningeal disease had responded, however, multiple sclerotic boney metastases had appeared in the cervical and thoracic spine; the patient was asymptomatic at the time. In February 2019, after 5 months of treatment, the patient had improvement in spinal leptomeningeal disease on MRI but worsening bony metastatic disease and marked intra-cranial progression (Fig. 3). Due to the observed clinical benefit, Sonidegib was continued and the patient received a course of palliative radiotherapy to the posterior fossa lesion (35 Gy in 10 fractions).

In April 2019, after a total of 6 months of Sonidegib treatment, the intracranial, leptomeningeal and bony metastatic disease all progressed, and all active treatment was ceased. The patient died 6 weeks later, 8.8 years from time of initial diagnosis.

## CASE 2

**Figure 4:**
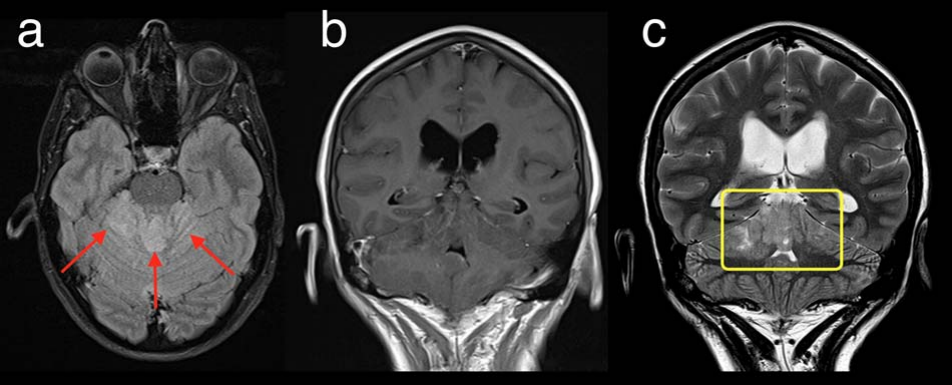
Axial T2 FLAIR (a), Coronal T1 post-contrast (b) and T2 (c) demonstrates a T2 FLAIR hyperintense mass lesion centred on the vermis (red arrows) in keeping with medulloblastoma. No enhancing component was identified (b) with heterogenous T2 signal within the vermis (yellow box).

A 56 year-old female presented in June 2017 with an 18-month history of mild ataxia and acute onset headaches. MRI revealed an ill-defined non-enhancing mass centred on the cerebellar vermis with local mass effect (Fig. 4). Due to the extent of disease and location, gross total resection was not feasible and the tumour was biopsied with posterior fossa suboccipital craniectomy decompression. Histopathology revealed a ‘classical’ medulloblastoma and molecular analysis was consistent with an SHH activated medulloblastoma via SMO mutation (p.W535L NM_005631 C.1604G > T) without TP53 mutation.

MRI spine showed no evidence of leptomeningeal spread and CSF analysis was clear. She was treated with three cycles of ICE, followed by cranio-spinal irradiation receiving 36 Gy in 20 fractions with a tumour cavity boost to 59.4 in 1.8 Gy/fraction. MRI brain on completion of radiotherapy, 5 months after post-operative imaging, showed significant volume reduction of 49% on T2 weighted imaging when compared with post-operative imaging (0.98 cm^2^ vs 1.89 cm^2^) with no enhancement seen on post-contrast imaging. At the patient’s request, adjuvant Sonidegib 200 mg daily was commenced in September 2018.

**Figure 5:**
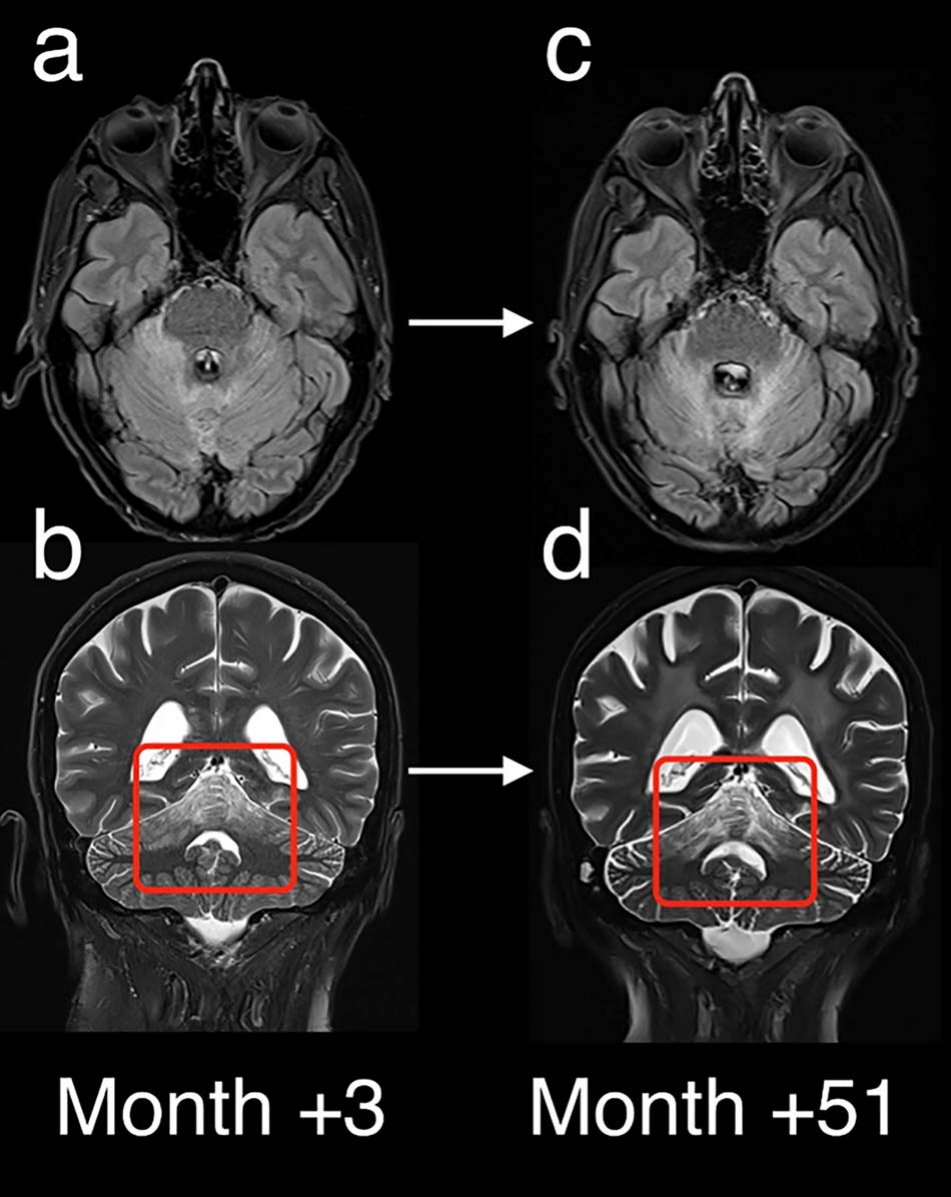
Long-term follow-up imaging with Axial T2 FLAIR (a/c) and Coronal T2 (b/d) shows stable T2 FLAIR signal and stable T2 signal with mild interval atrophy of the vermis (red box). No evidence of recurrence or progression at 51 months after chemoradiotherapy and adjuvant Sonidegib.

Although higher doses (usually 800 mg), are used in clinical trials, data from a basal cell carcinoma trial demonstrated equal efficacy between 200 mg and 800 mg daily [11]. Given this data and in the interest of limiting dose toxicity in the adjuvant setting, she remained on 200 mg daily for 14 months. Treatment was well-tolerated, with only CTCAE grade 2 alopecia and no severe adverse events. Ongoing surveillance shows no evidence of recurrence or progression on MRI 51 months following diagnosis (Fig. 5).

## DISCUSSION

Although rare, medulloblastomas in adults can cause significant morbidity and mortality. The role and timing of SHH inhibitors in managing this disease will need further study in clinical trials, however these will be limited by their low occurrence rate.

These two case reports demonstrate the drug was well-tolerated given at a dose of 200 mg daily. Case 1 showed leptomeningeal response after failing a number of cytotoxic agents and at a time of large tumour burden. Although the response was limited to intra-cranial disease, given this patient was heavily pre-treated, any response bares significance. In Case 2 Sonigedib was given in the adjuvant setting following chemotherapy and radiation. Due to limited surgery she is at high risk of recurrence, but remains disease free 51 months after diagnosis.

Both cases also showed potential clinical benefit of Sonidegib. The benefit criteria for Case 1 was the demonstrated transient improvement in leptomeningeal disease as well as clinical benefit in this heavily pre-treated patient. The benefit criteria for Case 2 is the ongoing progression free survival in the adjuvant setting for a high-risk patient in whom gross total resection could not be performed. To the authors’ knowledge, this is the first example of Sonigedib use in both these settings.

The use of Sonedigib in these settings warrants further investigation in the form of randomized control trials. This may enable its adoption into earlier lines of therapy in medulloblastoma treatment paradigms, with the hope of reducing the need for aggressive combination chemotherapy and cranio-spinal irradiation.
